# A word of caution on renal risks of prothrombin complex concentrate use in cardiac surgery

**DOI:** 10.1186/s13054-016-1254-0

**Published:** 2016-03-17

**Authors:** Christian J. Wiedermann

**Affiliations:** Department of Internal Medicine, Central Hospital of Bolzano/Bozen, Teaching Hospital of the Medical University of Innsbruck, Lorenz-Böhler Street 5, 39100 Bolzano, Bozen (BZ) Italy

In a retrospective study of bleeding patients in cardiac surgery, Cappabianca et al. [1] observed that the use of prothrombin complex concentrate (PCC) compared with fresh-frozen plasma (FFP) was associated with a higher risk of postoperative renal complications. In the unadjusted comparison, acute kidney injury (AKI) incidence was significantly higher in the PCC group, and use of PCC was an independent predictive factor of AKI development and the need for renal replacement therapy in the propensity-adjusted multivariate analysis. Since prothrombotic effects of PCC are unlikely as the underlying mechanism because an increase of thromboembolic events was not observed in the study, the authors speculate that volume excess given with FFP and a more hypovolemic balance with PCC in the context of bleeding patients could have exerted a protective effect on kidney function in the FFP group. Interestingly, in the PCC group, use of inotropes was significantly lower than in the FFP group (*p* = 0.007) [1]. This may have contributed to the observed difference in renal risk, since a recent meta-analysis on perioperative hemodynamic management associated inotropic drug use with a reduction in the incidence of postoperative AKI (odds ratio = 0.52, 95 % confidence interval = 0.34–0.80, *p* = 0.003, 14 studies, *n* = 1634 patients; included were two studies in cardiac surgery, *n* = 433 patients) [2]. The observed association of PCC use with higher risk of AKI may therefore be due to unknown confounders in this observational propensity score-matched analysis [3] rather than due to direct pharmacological effects of PCC.

## Abbreviations

AKI: acute kidney injury
FFP: fresh frozen plasma
PCC: prothrombin complex concentrate
