# Supplementary material for: A word of caution on renal risks of prothrombin complex concentrate use in cardiac surgery
Source: Crit Care. 2016 Mar 17;20:63. doi: 10.1186/s13054-016-1254-0 (PMC4794825; doi:10.1186/s13054-016-1254-0)
Supplement: Related texts

# Reply: Authors’ response

**Authors:** Giovanni Mariscalco, Daniele Maselli, Aamer B. Ahmed, Fausto Biancari

We read with great interest this letter from Wiedermann regarding our recent study [1] investigating the safety and efficacy of PCC as first-line treatment in bleeding after cardiac surgery. In our study, we observed an increased incidence of AKI and renal replacement therapy in the group of patients receiving PCC compared with those receiving FFP (31.3 % vs. 23.4 %, *p* = 0.01 and 3.1 % vs. 1.2 %, *p* = 0.037, respectively). We originally speculated that embolic events and especially the hypovolemia accompanying PCC use could have explained the observed correlation with renal dysfunction. Wiedermann underlined that the lower AKI rate observed in the FFP group could have been related to the high rate of inotropes (protective confounders) encountered in the same patient group rather than the hypovolemic balance following PCC use. We agree that postoperative AKI is a consequence of an interplay of different pathophysiologic mechanisms, and it remains distinctly possible that inotropes may protect against AKI, ameliorating blood pressure and perfusion [4]. In addition, our study could also have reflected the inherited inability of observational investigations in proving causality. However, the correlation between inotropes and AKI is disputed [5], and even in the meta-analysis by Prowle and colleagues [2, 5] the exact AKI contribution of inotropes and volume fluid resuscitation was difficult to ascertain. Nevertheless, our study did not show any significant difference between the rate of inotropes in the PCC and FFP propensity matched groups (odds ratio = 0.74, 95 % confidence interval = 0.50–1.08) [1].
